# Differential associations among social support, health promoting behaviors, health-related quality of life and subjective well-being in older and younger persons: a structural equation modelling approach

**DOI:** 10.1186/s12955-022-01931-z

**Published:** 2022-03-04

**Authors:** Phoenix K. H. Mo, Eliza L. Y. Wong, Nelson C. Y. Yeung, Samuel Y. S. Wong, Roger Y. Chung, Alan C. Y. Tong, Chris C. Y. Ko, Jia Li, Eng-kiong Yeoh

**Affiliations:** 1 Centre for Health Systems and Policy Research, JC School of Public Health and Primary Care, The Chinese University of Hong Kong, Shatin, Hong Kong; 2 Department of Psychology, The Chinese University of Hong Kong, Shatin, Hong Kong; 3 Faculty of Medicine, The University of New South Wales, Sydney, Australia

**Keywords:** Social support, Health-related quality of life, Health promoting behaviors, Younger persons, Older adults, Structural equation modelling

## Abstract

**Background:**

Extensive studies have confirmed social support as a critical protective factor of people’s health-related quality of life (HRQoL) and subjective well-being (SWB). However, health promoting behaviors as a potential mechanism and age differences in this mechanism has received fewer attention. This study aims to examine the associations among social support, health promoting behaviors, HRQoL and SWB in older and younger persons in Hong Kong.

**Method:**

A convenience sample of both younger (12–35 years old) and older persons (55 years old and above) were recruited from three non-government organizations to complete a survey. Structural Equation Model (SEM) was conducted to test both the measurement model and structural models to examine the relationship between social support, health promoting behaviors, HRQoL and SWB. Multi-group SEM was also performed and compared to test whether there were significant age differences in the pathways between the key variables.

**Results:**

A final sample of 408 participants (older-persons: N = 200 (mean age: 71.63 (8.16); 180/200 female), younger-persons: N = 208 (mean age: 18.10 (5.04); 155/208 female) were included in the final analysis. Results showed that social support was positively associated with SWB directly and indirectly through health promoting behaviors for the whole sample (CFI = .95, IFI = .94, RMSEA = .07, SRMR = 0.056). Results suggested that the association between the variables differed across age samples. While social support showed a positive association with health promoting behaviors for both younger and older persons, how each of them associated with HRQoL and SWB was different.

**Conclusion:**

Findings suggest that the pathway which social support linked with HRQoL and SWB might differ across age groups. Age-specific strategies should be considered when promoting HRQoL and SWB among the younger and older population.

**Supplementary Information:**

The online version contains supplementary material available at 10.1186/s12955-022-01931-z.

## Introduction

Health-related quality of life (HRQoL) is a multi-dimensional concept that is related to an individual’s physical, mental, and social functioning [1, 2]. It is a broad term involving different perspectives of an individual’s life. A related and equally important concept is subjective well-being (SWB), which includes positive self-evaluations and affective reactions to one’s experiences [3]. Both HRQoL and SWB have been used as key indicators of health of a population [2, 4]. Being different from traditional quantifiable indicators (e.g. mortality and morbidity), HRQoL and SWB depict the overall impression of a patient’s condition as they represent the endpoint most pertinent to the person [4, 5]. In terms of disease prevention, HRQoL and SWB are important factors for monitoring individuals’ health status. Studies among the disease populations have also shown that HRQoL and SWB were closely related to clinical indicators, morbidity, mortality, and survival [6–8]. One study among a representative sample of 1,681 older persons in Chile found that lower perception of quality of life (QoL) was a significant predictor of five-year mortality [9].

The important role of HRQoL and SWB has been considered and documented extensively in both the younger and older populations. In a chronic disease setting, HRQoL and SWB are the prime focus of treatment and care [10]. As an individual ages, their HRQoL and SWB may also change with their life events and personal development during the transition. It is therefore important to examine the HRQoL and SWB among individuals in different stages of life, and to explore if their associated factors may differ across individuals in different age.

### Influence of social support on HRQoL and SWB

HRQoL and SWB can be affected by a range of personal, psychosocial, and behavioral factors [11]. Among all the social determinants of health, social support is a well-documented protective factor for people’s HRQoL and SWB. For instance, studies have also shown that support and satisfaction with the community were associated with better community SWB and HRQoL [12, 13]. One study among older persons receiving CBT treatment showed that positive change in social support was associated with improvement in QoL beyond the effects of the CBT treatment [14].

Social support is broadly defined as one’s perception that they are being cared for and that assistance would be available when needed [15, 16]. It is mainly provided by family members, close friends, or the community [17]. Theoretical models, namely social network and social capital, have attempted to explain the mechanisms regarding how social capital can influence health. Specifically, social support helps to buffer stress, provide protective psychological resources (e.g., self-control, self-efficiency, mastery), and health-promoting norms and attitudes to promote health behaviors and outcomes [18, 19].

### Influence of social support on HRQoL and SWB: health promoting behaviors as a mediator

Despite the numerous studies linking social support with health and well-being, there are limited studies examining health behaviors specifically as a potential mechanism in-between. Health promoting behavior refers to self-initiated and continuous activities undertaken for the purpose of increasing or promoting an individual’s health and SWB [20]. Healthy lifestyles can help prevent chronic diseases such as cardiovascular diseases, cancer and mental health illness via biological and psychological mechanisms such as reversing cell aging and buffering stress, and therefore contribute to HRQoL [21]. A World Health Organization (WHO) cross-nation study of 35 countries found that lifestyle factors predicted about 60% of HRQoL among individuals [22]. There is ample evidence that individuals who engaged in health promoting behaviors would report better health and lower levels of disease and morbidity [23, 24]. Health promoting behaviors can also boost SWB by generating autonomy, mastery, meaning and affiliation [25]. Research among various populations has consistently shown that healthy lifestyle, such as moderate level of physical activity, healthy eating pattern, lower level of screen time, and stress management, is associated with better HRQoL and SWB [26–29]. Studies among community-dwelling Chinese older persons [30] and Chinese university students [31] documented that health promoting lifestyles were associated with better psychosocial SWB and lower depression.

Empirical evidence has shown that, social support may influence an individual physically and psychologically, which may initiate favorable health actions such as physical activity, diet, and compliance to medical treatments [19, 32, 33]. For instance, smokers receiving and perceiving extensive partner support have a higher rate of cessation or short-term withdrawal from smoking [34]. Other studies have also shown that people who are married [35, 36], have a larger social network [37, 38], or involved in religious communities [39] have a higher chance of adopting optimal health behaviors. One study of 941 adults in Hong Kong found that social support was associated with mental health promoting behaviors, which in turn was associated with better HRQoL and mental health [40]. There is also evidence that intervention that promoted social support were effective in improving healthy lifestyle and HRQoL among patients with chronic kidney disease [41].

### Differential association among social support, health promoting behaviors, HRQoL and SWB in older and younger persons

Social support and health promoting behaviors may play different roles in life courses. However, few studies to date have examined the potential age differences in the associations between these variables. Examining whether the association between social support, health promoting behaviors, HRQoL and SWB differ among across the older and younger sample will be necessary to develop interventions and policies in promoting health for people with different ages. The Socioemotional Selectivity Theory [42, 43] proposes that individuals’ social goals change with age. Older individuals are more likely to focus on satisfying social and emotional goals and devote their time and energy on intimate social contacts, such as close friends and family. With strong ties being the main source of social support, older persons might be more likely to prioritize social support and perceive is as an important contributing factor to their SWB and HRQoL. A national survey among people ages 65 in Britain reported that social relationship was the most commonly cited (81%) component to their QoL [44]. Social support might have a stronger association with HRQoL and SWB in the older sample compared to the younger one.

### The present study

The present study examined the association among social support, health promoting behaviors, HRQoL, and SWB in older and younger persons in Hong Kong. It is hypothesized that social support is positively associated with health promoting behaviors, which are positively associated with HRQoL and SWB. Social support also has direct positive associations with HRQoL and SWB. It is also hypothesized that the association between the variables will be stronger among the older population sample.

## Methods

### Participants and procedures

Participants were recruited from three local non-government organizations (NGOs) that provided community-based services to older and younger persons. Inclusion criteria for the older participants were 55 years old or above, intellectually capable, and able to comprehend Cantonese. Those who were diagnosed with dementia and any mental disorders, or unable to communicate verbally were excluded from the study. Inclusion criteria for the younger persons were aged between 12 and 35 years old, while other inclusion and exclusion criteria remained identical to those of the older persons’ group.

The staff of the NGOs approached prospective participants and interested participants were invited to meet the research assistant at the NGO. The purpose and logistic of the study were first explained, then participants were assured that the study was voluntary, refusal to join the study would not affect any future services they would use, and they could withdraw from the study anytime. Those who agreed to take part in the study were asked to provide written informed consent. Parents’ consents were also sought for participants who were under 18 years old. The parents were fully informed the objective of the study and also the details of the questionnaire. After obtaining informed consent, participants were invited to fill in a questionnaire which took around 20 min to complete. Participants who were illiterate were assisted by research assistant to complete the survey. Ethics approval was obtained from Survey and Behavioral Research Ethics Board at the authors’ institution (Ethics ID: S66500309).

### Measurements

#### Perceived social support

The support subscale from the Comprehensive Inventory of Thriving (CIT) [45] was adopted to assess participants’ level of social support. The original subscale contains three items which measure the level of social support participants received in general. The subscale has been translated and validated in Chinese sample [46]. In the present study, the three items were expanded into six items, in which three items measured level of social support received from family and relatives, and three other items measured level of social support received from friends and community members. Items were rated on a 5-point Likert Scale from 1 (strongly disagree) to 5 (strongly agree). The Cronbach alpha of the scale was 0.93. The two subscales, social support from family and relatives, and social support from friends and community members served as parcel indicators of social support in the present study. The overall score was averaged and ranged from 1 to 5 with higher score indicating a higher level of social support.

#### Health-promoting behaviors

Health promoting behaviors were measured by four items extracted from the Health Promoting Lifestyle Profile questionnaire [20]. The scale has been tested and validated among Chinese population [47]. One item each from the following subscale was extracted from the questionnaire: (1) Physical activity (i.e., exercised for three times a week), (2) Nutrition (i.e., followed a 3-low-1-high, i.e. low fat, low sugar, low salt, high fibre diet regime), (3) Interpersonal relations (i.e., maintained social contact with acquaintances), and (4) Stress management (i.e., applied any strategy to cope with stress), respectively. Items were rated on a 5-point Likert scale ranged from 1 (never) to 5 (always). The Cronbach alpha of the scale was 0.65. The four items served as parcel indicators of health-promoting behaviors in the present study. The final score ranged from 1 to 5 with higher score indicating better health lifestyles.

#### HRQoL

The EQ-5D-5L (EuroQol) [48] was used to measure participants’ health-related quality of life (HRQoL). The validated Hong Kong Chinese version was used in this study [49]. The EQ-5D-5L consists of two parts, namely the EQ descriptive system and the EQ visual analogue scale (EQ VAS). For the EQ-5D descriptive system, respondents indicated their health status on five dimensions: mobility, self-care, usual activities, pain/discomfort, and anxiety/depression. Each dimension was rated on five levels: no problems, slight problems, moderate problems, severe problems, and extreme problems. A locally validated algorithm was used to convert the rating into a single summary index. Possible score on this index ranged from -0.8637 (worse heath) to 1.0 (full health) [50]. The Cronbach alpha of the scale was 0.67. For the EQ VAS, participants were asked to self-rate their health on a vertical visual analogue scale, where the endpoints are labelled ‘the best health you can imagine’ (score of 100) and ‘the worst health you can imagine’ (score of 0). The EQ VAS was used as a quantitative measure of HRQoL with a score of 1 to 100 (totally healthy) that reflected the participants’ own judgement. The EQ descriptive system and EQ VAS served as parcel indicators of HRQoL in the present study.

#### SWB

The 3-item positive feelings subscale and one item from the life satisfaction (i.e. I am satisfied with my life) subscale from the CIT [45] were adopted to examine the participants’ level of SWB. The CIT has been tested and validated in Chinese population [46]. Items were rated on a 5-point Likert Scale from 1 (strongly disagree) to 5 (strongly agree). The Cronbach alpha of the scale was 0.89. The four items served as parcel indicators of SWB in the present study. The overall score ranged from 1 to 5 with higher score indicating better SWB.

## Data analysis

Descriptive statistics for the older and younger persons’ samples were explored. The differences in the variables under study (i.e., social support, health promoting behaviors, HRQol, and SWB) between the two sub-samples were examined using independent sample t-tests. To examine the associations among social support, health promoting behaviors, HRQoL and SWB, confirmatory factor analysis (CFA) was first conducted to evaluate the measurement model [51]. Structural equation modelling (SEM) was then employed to test the structural model for the whole sample [52]. Normality tests were performed by examining the skewness and kurtosis of all the outcome variables including social support, health promoting behaviours, SWB and HRQoL. Except for the two constructs of HRQoL, all other variables were normally distributed with both the kurtosis and skewness less than 2. Due to the skewed nature of HRQoL instruments, we adopted the robust maximum likelihood method as the estimator which can be applied to non-normally distributed data in the SEM analysis [53]. Multi-group analysis was then performed to compare two structural models within the analysis: a restricted model with all parameters estimated to be equal across age groups, and an unrestricted model which all parameters estimated were allowed to differ across groups. To examine the significance of each path across groups, a series of models with different paths being constrained were also compared. All data analyses were performed using RStudio [54]. The R syntax of the abovementioned structural SEM and multi-group SEM model under different conditions was attached as Additional file 1: Appendix.

*G*oodness-of-fit indices, including incremental fit index (IFI), comparative fit index (CFI), root mean square error of approximation (RMSEA), and Standardized Root Mean Square Residual (SRMR) were used to evaluate the model fit. IFI and CFI range between 0 and 1, and values over 0.90 indicate a good fit [55]. A RMSEA and SRMR value with less than 0.05 means an excellent fit whereas a value between 0.05 and 0.08 reflects a reasonable fit [56]. The R syntax of these SEM models to calculate the model fit indices (“*fitMeasures*” function) and compare indices of different models (“*compareFit*” function) is also shown in the Additional file 1: Appendix. The sample size for the whole analysis is considered sufficient based on the widely used 10-time rules that the sample size should be greater than the maximum number of model links to a latent variable in the model [57]. The significance level of the whole analysis was set as 0.05.

## Results

### Descriptive statistics

In the period from April 2018 to March 2019, we collected 408 valid questionnaires (N = 200 for the older persons’ sample, N = 208 for the younger persons’ sample). The sociodemographic characteristics of the participants are presented in Table 1. The mean age of the overall sample is 44.31 (SD = 27.58). For the older adults’ sample, their mean age was 71.56 years old (range: 59–94; SD = 8.00). Majority of the participants were female (90.0%) and had a primary or below level of education (85.0%). Slightly more than half of them (56.5%) were married, and 37.0% of them reported having a monthly household income of HKD 10,000 or below. For the younger persons’ sample, their mean age was 18.10 years old (range: 12 to 35; SD = 5.04). 123 of them were adolescents under 18 years old. For robustness check, we conducted the same analysis with the adult sample (≥ 18 years old) as well and the results were similar with the whole sample (see Additional file 1: Tables S1 and S2 for the results of the SEM models). More than two-third of the participants were female (74.5%) and had a primary or below level of education (71.6%). All except one participant were single (99.5%).

**Table 1:** Sociodemographic characteristics of the sample (N = 420)

	Elderly (N = 200)	Youth (N = 208)	Difference between groups
	M (SD) / N (%)	M (SD) / N (%)	
Mean age (years)	M = 71.63 (8.16)	M = 18.10 (5.04)	N.A
**Gender**			
Male	20 (10.0%)	53 (25.5%)	N.A
Female	180 (90.0%)	155 (74.5%)	
**Highest level of education obtained**			
Primary or below	170 (85.0%)	149(71.6%)	N.A
Secondary	12 (6.0%)	3 (1.4%)	
Diploma	6 (3.0%)	17 (8.2%)	
University or above	12 (6.0%)	39 (18.8%)	
**Marital status**			
Single	20 (10.0%)	207 (99.5%)	N.A
Married	113 (56.5%)	1 (0.5%)	
Widowed / divorced / separated	67 (33.5%)	0 (0.0%)	
**Monthly household income (in HKD)**			
$10,000 or below	74 (37.0%)	20 (9.6%)	N.A
$10,001 to $20,000	27 (13.5%)	27 (13.0%)	
$20,001 to $30,000	9 (4.5%)	31 (14.4%)	
$30,001 to $40,000	3 (1.5%)	12 (5.8%)	
$40,001 to $60,000	8 (4.0%)	19 (9.1%)	
$60,001 or above	7 (3.5%)	27 (13.0%)	
Unknown	72 (36.0%)	73 (35.1%)	
Social support	M = 3.80 (.71)	M = 3.85 (.75)	*t*(406) = − .79
Health promoting behaviors	M = 3.93 (.66)	M = 3.42 (.66)	*t*(406) = 7.90***
SWB	M = 3.97 (.62)	M = 3.67 (.69)	*t*(406) = 4.67***
HRQoL—EQ Index	M = .83 (.18)	M = .94 (.10)	*t*(406) = − 7.42***
HRQoL—EQ VAS	M = 80.85 (13.23)	M = 86.09 (12.31)	*t*(406) = -4.14***

*NA* not applicable, *SWB* subjective well-being, *HRQoL* health-related quality of life

***p < .001

The mean score of the variables under study is presented in Table 1. Results from independent sample t-tests showed that the older adults sample scored significantly higher in health promoting behaviors, *t*(406) = 7.90, *p* < 0.001 and SWB, *t*(406) = 4.67, *p* < 0.001 than their younger counterparts. On the other hand, they scored significantly lower in HRQoL (*t*(406) = −7.42 for EQ Index and *t*(406) = −44.14 for EQ VAS) than the younger counterparts.

### Measurement model of the hypothesized model for whole sample

Table 2 presents the factor loadings of the measurement model for the whole sample. The measurement model yielded a good fit, CFI = 0.95, IFI = 0.94, RMSEA = 0.07, SRMR = 0.056. Standardized factor loading of the measurement model ranged from 0.41 to 0.92 and were all statistically significant at the *p* < 0.001 level.

**Table 2:** Unstandardized and Standardized Loadings for the measurement model

Parameter estimates	Standardized loading
Social support → Social support from family and relatives	.78
Social support → Social support from friends and community members	.80
Health promoting behaviors → Item 1	.50
Health promoting behaviors → Item 2	.48
Health promoting behaviors → Item 3	.69
Health promoting behaviors → Item 4	.57
SWB → Item 1	.69
SWB → Item 2	.87
SWB → Item 3	.92
SWB → Item 4	.80
HRQoL → EQ descriptive system	.41
HRQoL → EQ VAS	.86

### Structural model of the hypothesized model

**Fig. 1:**
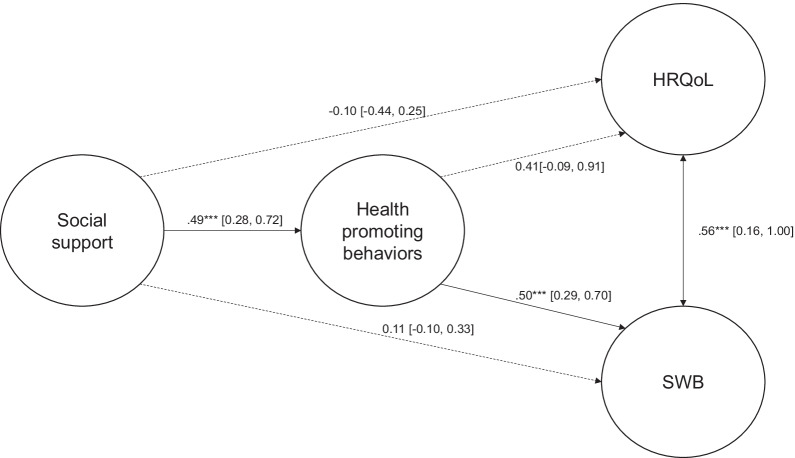
Structural model of social support, health promoting behaviors, health-related quality of life, and subjective well-being for the whole sample. *p < .05, **p < .01, *** *p* < .001

Results of structural equation modelling showed that the proposed model for the whole sample yielded a good fit (CFI = 0.95, IFI = 0.94, RMSEA = 0.07, SRMR = 0.056). In sum, social support was positively associated with health promoting behaviors (*β* = 0.44, 95% CI = [0.25, 0.63], *p* < 0.001), and SWB (*β* = 0.20, 95% CI = [0.04, 0.35], *p* < 0.05). Health promoting behaviors were positively correlated with SWB (*β* = 0.42, 95% CI = [0.27, 0.57], *p* < 0.001). The standardized path coefficients of the structural model are shown in Fig. 1.

### Multi-group analyses by sample

Results of the multi-group analysis of the measurement model showed that the unrestricted model showed a significantly better model fit (Models 1.1 and 1.2 of Table 3). Therefore, to examine the equality of the structural model across sample, all factor loadings were estimated freely.

**Table 3:** Summary statistics for tested models in multi-group analyses

	CFI	IFI	RMSEA	SRMR
**Measurement model**				
Model 1.1	.98	.97	.05	0.05
Model 1.2	.90	.89	.09	0.10
**Structural model**				
Model 2.1	.98	.97	.04	0.05
Model 2.2	.87	.85	.10	0.11
Model 2.3	.90	.89	.09	0.10
Model 2.4	.87	.85	.10	0.11
Model 2.5	.93	.91	.07	0.08
Model 2.6	.88	.87	.09	0.10

1.1 All factor loadings in the measurement model were estimated freely

1.2 All factor loadings in the measurement model were constrained to be equal

2.1 All path coefficients in the structural model were estimated freely

2.2 Path coefficient from social support to health promoting behaviors was constrained to be equal

2.3 Path coefficient from social support to HRQoL was constrained to be equal

2.4 Path coefficient from social support to SWB was constrained to be equal

2.5 Path coefficient from health promoting behaviors to HRQoL was constrained to be equal

2.6 Path coefficient from health promoting behaviors to SWB was constrained to be equal

Results of the multi-group analysis of the structural model showed that constraining either path in the model resulted in a significant change in model fit. Overall, comparison of the various models showed that Model 2.1, in which all parameters were estimated freely, showed the best fit to the model, CFI = 0.98, IFI = 0.97, RMSEA = 0.05, SRMR = 0.05) indicating that the association among social support, health promoting behaviors, HRQoL, and SWB differed across sample. Further analyses revealed that social support showed significant association with HRQoL (*β* = 0.25, 95% CI = [0.01, 0.49], *p* < 0.05) and SWB (*β* = 0.37, 95% CI = [0.12, 0.62], *p* < 0.01). Social support also had a significant association with health promoting behaviors in the older persons’ sample (*β* = 0.45, 95% CI = [0.16, 0.75], *p* < 0.01) Furthermore, health promoting behaviors showed no significant association with HRQoL and SWB in the older persons’ sample.

**Fig. 2:**
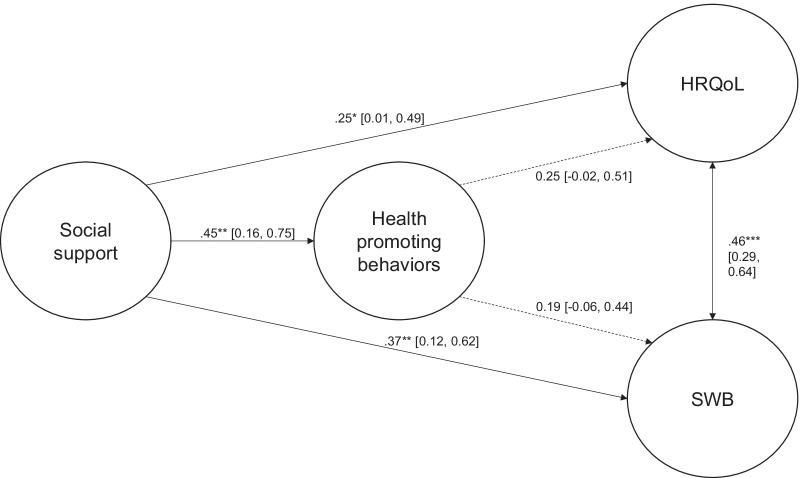
Structural model of social support, health promoting behaviors, health-related quality of life, and subjective well-being for the older persons. *p < .05, **p < .01, *** *p* < .001; Dashed lines indicate non-significant relationships

**Fig. 3:**
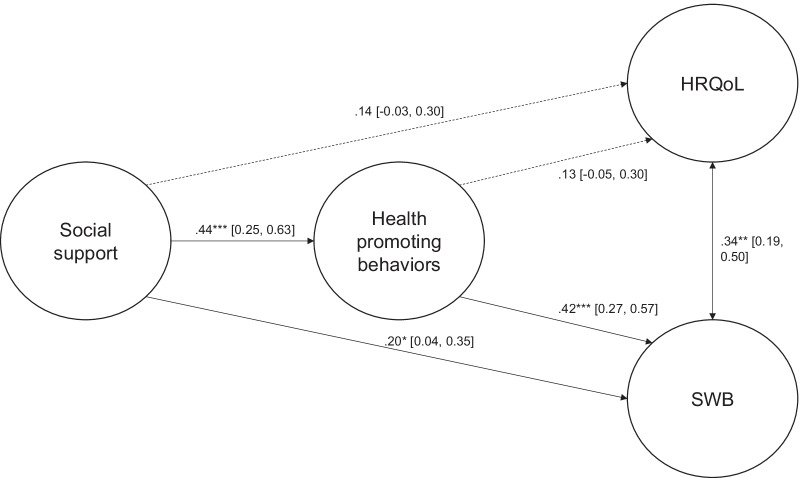
Structural model of social support, health promoting behaviors, health-related quality of life, and subjective well-being for the younger persons. *p < .05, **p < .01, *** *p* < .001; Dashed lines indicate non-significant relationships

In the younger persons’ sample, social support showed a significant positive association with health promoting behaviors (*β* = 0.49, 95% CI = [0.28, 0.72], *p* < 0.001), which in turn was positively associated with SWB (*β* = 0.50, 95% CI = [0.29, 0.70], *p* < 0.001) but not with HRQoL. However, social support also showed no significant associations with either SWB or HRQoL. Figures 2 and 3 present the standardized path coefficients and 95% CI of the structural model for the older and younger persons’ samples respectively.

## Discussion

The present study examined the association among social support, health promoting behaviors, HRQoL, and SWB in older and younger persons in Hong Kong. In the whole sample, social support was directly associated with higher SWB. This is consistent with previous literature in the regard that how social support can act as instrumental and emotional resources for people to achieve better health outcomes [18, 19]. High levels of perceived social support might have a positive influence on psychosocial functioning and protection from mental health problems [58, 59]. Social support may boost one’s feelings of respect, self-worth, and dignity, which contributes to better SWB [19]. They may also have more emotional or cognitive resources in meeting challenges or being more open to new challenges, which promote independence. Those with promising support from the community may also be more likely to demonstrate satisfaction with community services and conditions, which promote SWB [12].

The importance of social support is further emphasized in its indirect association with SWB through health promoting behaviors. Social support may also drive individuals into health promoting behaviors in different ways [60]. For example, participants’ friends, family, and supportive others can provide informative and tangible forms of support, model positive health behaviors and promote mastery, provide encouragement for engaging in positive behaviors, or motivate them by showing that barriers to the health promoting behaviors can be overcome [61–63]. They may also assist the individuals in attaining their health-related goals, or serve as agents of social control to positively influence health behaviors [64]. Health promoting behaviors were associated with higher SWB in the whole sample. These were consistent with the existing literature that some of the health promoting behaviors, e.g., physical activity, stress management, and managing interpersonal relationships, were also found to be linked to boosts in SWB [65, 66]. Health promotion behaviors, such as physical activities, can fulfill one’s psychological needs such as generating autonomy, mastery, meaning and affiliation, leading to better SWB.

However, in the whole sample, neither social support nor health promoting behaviors had direct associations with HRQoL. A potential explanation is that over a half of the whole sample is younger people aged under 35 and there might be very little variations in their HRQoL due to their young age. HRQoL, compared to SWB, is more of an objective measure of their health conditions and therefore younger people’s health may not be significantly influenced by social support or health promoting behaviors.

It is important to note a robustness check was conducted with the adult-only sample (≥ 18 years old) and the results were similar with the whole sample; the robustness of the results can thus be assumed.

### Differential associations between variables across samples

While positive associations among social support, health promoting behaviors and SWB were observed in the whole sample, the present study also found that the associations between these variables varied across age groups. While social support was significantly protective of health promoting health behaviors in both younger and older groups, older participants’ HRQoL and SWB were mainly predicted by social support, which is not the case among younger people. Such findings were in line with the literature that demonstrated social relationships as the most important source of health and well-being among the older persons, according to the socio-emotional selectivity theory [44].

Health promoting behaviors were found in explaining better SWB among the younger persons’ but not the older persons’ sample. It is consistent with previous literature regarding young people’s lifestyle and their SWB. On the other hand, health promoting behaviors had no association with either HRQoL or SWB in the older sample. There could be several explanations. Adolescence is the beginning of developmental stage when individuals enjoy more autonomy with their decision making with regards to their health [67]. It may be plausible that the younger participants are at the stage when they need to learn the skills to formulate their own healthy lifestyles and maintain such habits into adulthood. They might therefore be more likely to appreciate the benefits of social support on health promoting behaviors and the important role of health promoting behaviors on SWB, and have attributed their better SWB to healthy lifestyle. By contrast, older adults may have some pre-existing health behaviors which may not be considered “healthy”, and sticking to health promoting behaviors may not necessarily mean higher SWB and instead, may be a compromise of their preferred lifestyle for the sake of better physical condition. The differences between the younger and older sample in this study indicated that the associations between social support and people’s health and well-being differ by age. While social support can promote young people’s well-being via health promoting behaviors, for older people, there may probably be other mechanisms.

### Implications

Findings of the present study have important implications for public health practice. Healthy lifestyle is a key element in disease prevention and better public health. Based on the social determinants of health framework, promoting healthy lifestyle has been initiated in the governmental policies of some countries, such as China, to improve population health [68]. SWB, featured by happiness and an individual’s own evaluation of life, should be put on policy agenda to inform public health decision-making [69]. This study provided some preliminary evidence consistent with the social determinants of health by highlighting the importance of social support in shaping both health behaviors and SWB. It requires the public health policy to take into consideration the need to foster social connection and social support among people, especially for those who face higher risk of social isolation (e.g., older adults who are widowed, disabled, or living alone etc.) to reduce health inequality [70, 71]. It may be particularly important for older persons, as they are more likely to experience social isolations and health declines [72]. Social support interventions could help individuals expand the existing social network so as to increase the emotional, informational, or tangible support that one could receive. Health care professionals could also improve the quality of social support from the individuals’ existing networks by equipping individuals with the skills to improve quality of social interactions, to seek for supportive help when needed, to reduce interpersonal conflicts, and to change negative perceptions of others [73].

While ample evidence has shown that encouraging health-promoting lifestyle can help promote or maintain HRQol and SWB, findings of the present study call for special attention when considering the promotion of healthy lifestyle as its effect on HRQol and SWB was not evident among the older persons. Given the significant and direct association between social support, HRQol and SWB among the older persons, social support should be highly emphasized in health promotion for them. Whereas for the younger persons sample, health promoting behaviors contribute substantially to the association between social support and SWB. Facilitating uptake of health promoting behaviors may be a suitable target for interventions aimed at promoting SWB for the young persons, Social support should also be emphasized, so that individuals are empowered within a supportive atmosphere that can facilitate their uptake and maintenance of health promoting behaviors.

### Limitations

The present study is subject to some limitations. First, the study was cross-sectional in nature so causal inferences cannot be made. Recent studies have also documented the impacts of health on social capital and this calls for future studies to more closely examine the potentially bi-directional relationship between health and its social determinants [74]. However, it is important to note that the hypothesized relationships between the variables in the present study made theoretical sense. Longitudinal or experimental studies in the future should be conducted to examine the causal relationship between social support, health-related behaviors and health outcomes. For instance, randomized controlled trials could be developed to test the effectiveness of interventions regarding social support in changing people’s health behaviors and consequential outcomes. Second, data were measured using self-report which is subject to recall bias and over-reporting/underreporting. Third, participation was voluntary in nature, those who agreed to take part in the study might have better health and were more likely to engage in healthy lifestyles. Fourth, it is expected that participants’ HRQoL and SWB would also be affected by their medical background (e.g. presence of chronic conditions) but such information was not obtained in the study. Fifth, participants were recruited from only three NGOs in Hong Kong so the findings might not be generalizable to the general population. Sixth, participants were predominantly female and due to the unbalanced distribution of gender in our sample, we were not able to examine potential gender differences in the associations. Future studies should collect larger and more balanced sample to investigate potential gender differences.

## Conclusion

The present study revealed differential associations among social support, health promoting behaviors, HRQoL, and SWB between the younger and older persons in Hong Kong, which provide important implications for public health services. Social support was directly associated with SWB and HRQoL among older persons instead of younger persons. Social support indirectly associated with SWB through health promoting behaviors among the younger persons, but not the older persons. Age-specific strategies should be considered when promoting HRQoL and SWB, and prospective studies are warranted to confirm the causal relationships among the variables.

## Supplementary Information

**Additional file 1**. Tables S1 and S2 for the results of the SEM models.

## Data Availability

The datasets generated and/or analysed during the current study are not publicly available due to privacy or ethical restrictions but are available from the corresponding author on reasonable request.
