# Supplementary material for: Differential associations among social support, health promoting behaviors, health-related quality of life and subjective well-being in older and younger persons: a structural equation modelling approach
Source: Health Qual Life Outcomes. 2022 Mar 4;20:38. doi: 10.1186/s12955-022-01931-z (PMC8895671; doi:10.1186/s12955-022-01931-z)
Supplement: Supplementary file 1 — Additional file 1. Tables S1 and S2 for the results of the SEM models. [file 12955_2022_1931_MOESM1_ESM.docx]

**Appendix**

**Table 1 Structural model of the whole adult sample**

| Pathway |  |  |  | Beta | SE | z | p-value | 95% LL | 95% UL |
| --- | --- | --- | --- | --- | --- | --- | --- | --- | --- |
| HRQoL | ~ | Social Support | b2 | 0.147 | 0.114 | 1.283 | 0.200 | -0.078 | 0.371 |
| HRQoL | ~ | Health promoting behaviours | c2 | 0.103 | 0.103 | 0.996 | 0.319 | -0.099 | 0.305 |
| SWB | ~ | Social Support | b1 | 0.212 | 0.101 | 2.102 | 0.036 | 0.014 | 0.409 |
| SWB | ~ | Health promoting behaviours | c1 | 0.33 | 0.091 | 3.645 | 0.000 | 0.153 | 0.508 |
| Health promoting behaviours | ~ | Social Support | a | 0.393 | 0.147 | 2.679 | 0.007 | 0.105 | 0.681 |
| SWB | ~~ | HRQoL |  | 0.42 | 0.08 | 5.269 | 0.000 | 0.264 | 0.576 |
| CFI |  | 0.953 |  |  |  |  |  |  |  |
| IFI |  | 0.946 |  |  |  |  |  |  |  |
| RMSEA |  | 0.063 |  |  |  |  |  |  |  |
| SRMR |  | 0.058 |  |  |  |  |  |  |  |

**Table 2 Multi-group structural model of the whole adult sample**

| Pathway |  |  |  |  | Beta | SE | z | p-value | 95% LL | 95% UL |
| --- | --- | --- | --- | --- | --- | --- | --- | --- | --- | --- |
| HRQoL | ~ | Social Support | Older | b1 | 0.25 | 0.123 | 2.036 | 0.042 | 0.009 | 0.491 |
| HRQoL | ~ | Health promoting behaviours | Older | c1 | 0.249 | 0.135 | 1.84 | 0.066 | -0.016 | 0.514 |
| SWB | ~ | Social Support | Older | b2 | 0.367 | 0.127 | 2.888 | 0.004 | 0.118 | 0.617 |
| SWB | ~ | Health promoting behaviours | Older | c2 | 0.194 | 0.127 | 1.521 | 0.128 | -0.056 | 0.444 |
| Health promoting behaviours | ~ | Social Support | Older | a | 0.452 | 0.15 | 3.019 | 0.003 | 0.159 | 0.745 |
| HRQoL | ~ | Social Support | Younger | bb1 | -0.227 | 0.161 | -1.408 | 0.159 | -0.543 | 0.089 |
| HRQoL | ~ | Health promoting behaviours | Younger | cc1 | 0.351 | 0.195 | 1.796 | 0.072 | -0.032 | 0.733 |
| SWB | ~ | Social Support | Younger | bb2 | -0.068 | 0.158 | -0.431 | 0.666 | -0.378 | 0.241 |
| SWB | ~ | Health promoting behaviours | Younger | cc2 | 0.323 | 0.189 | 1.715 | 0.086 | -0.046 | 0.693 |
| Health promoting behaviours | ~ | Social Support | Younger | aa | 0.534 | 0.155 | 3.451 | 0.001 | 0.231 | 0.838 |
| CFI | 0.983 |  |  |  |  |  |  |  |  |  |
| IFI | 0.970 |  |  |  |  |  |  |  |  |  |
| RMSEA | 0.036 |  |  |  |  |  |  |  |  |  |
| SRMR | 0.052 |  |  |  |  |  |  |  |  |  |

##structural model

structural_model <-'

##measurement model

support=~pre_csupport+pre_fsupport

health_beha=~pre_lifestyle2+pre_lifestyle3+pre_lifestyle4+pre_lifestyle5

swb=~pre_sub_wellbeing1+pre_sub_wellbeing2+pre_sub_wellbeing3+pre_sub_wellbeing4

HRQoL=~pre_EQ5D_index+pre_EQ5D_VAS_log

##regression

HRQoL~b2*support+c2*health_beha

swb~b1*support+c1*health_beha

health_beha~a*support

##residual correlation

HRQoL~~swb

'

structural_model<-cfa(structural_model,data=data,std.lv = TRUE,estimator="mlr")

summary(structural_model,standardized=TRUE,rsquare=T)

fitMeasures(structural_model,c("chisq","chisq.scaled","df","cfi","cfi.robust","ifi","ifi.scaled","rmsea","rmsea.robust","srmr"))

standardizedsolution(structural_model)

##multigroup structural model

structural_model <-'

##measurement model

support=~pre_csupport+pre_fsupport

health_beha=~pre_lifestyle2+pre_lifestyle3+pre_lifestyle4+pre_lifestyle5

swb=~pre_sub_wellbeing1+pre_sub_wellbeing2+pre_sub_wellbeing3+pre_sub_wellbeing4

HRQoL=~pre_EQ5D_index+pre_EQ5D_VAS_log

##regression

HRQoL~c(b1,bb1)*support+c(c1,cc1)*health_beha

swb~c(b2,bb2)*support+c(c2,cc2)*health_beha

health_beha~c(a,aa)*support

##residual correlation

HRQoL~~swb

##indirect

ab1 := a*c1

ab2 := a*c2

aacc1 := aa*cc1

aacc2 := aa*cc2

'

#free model

structural_model_multi<-cfa(structural_model,data=data,std.lv = TRUE,group = "Generation",estimator="mlr")

summary(structural_model_multi,standardized=TRUE,rsquare=T)

fitMeasures(structural_model_multi,c("chisq.scaled","df","cfi.robust","ifi","rmsea.robust","srmr"))

standardizedsolution(structural_model_multi)

##equal all

structural_model_constrain1<-cfa(structural_model,data=data,std.lv = TRUE,group = "Generation",group.equal=c("intercepts","regressions","loadings","residuals"),estimator="mlr")

summary(structural_model_constrain,standardized=TRUE)

fitMeasures(structural_model_constrain,c("chisq.scaled","df","cfi.robust","ifi","rmsea.robust","srmr"))

standardizedsolution(structural_model_constrain)

##free different paths

structural_model_constrain1 <-

cfa(

structural_model,

data = data,

std.lv = TRUE,

group = "Generation",

group.equal = c("loadings", "intercepts", "regressions","residuals"),

estimator = "mlr"

)

structural_model_constrain2 <-

cfa(

structural_model,

data = data,

std.lv = TRUE,

group = "Generation",

group.equal = c("loadings", "intercepts", "regressions"),

estimator = "mlr"

)

structural_model_constrain3 <-

cfa(

structural_model,

data = data,

std.lv = TRUE,

group = "Generation",

group.equal = c("loadings", "intercepts", "residuals"),

estimator = "mlr"

)

structural_model_constrain4 <-

cfa(

structural_model,

data = data,

std.lv = TRUE,

group = "Generation",

group.equal = c("loadings", "regressions","residuals"),

estimator = "mlr"

)

structural_model_constrain5 <-

cfa(

structural_model,

data = data,

std.lv = TRUE,

group = "Generation",

group.equal = c("intercepts", "regressions","residuals"),

estimator = "mlr"

)

fitMeasures(structural_model_constrain1,c("chisq.scaled","df","cfi.robust","ifi","rmsea.robust","srmr"))

fitMeasures(structural_model_constrain2,c("chisq.scaled","df","cfi.robust","ifi","rmsea.robust","srmr"))

fitMeasures(structural_model_constrain3,c("chisq.scaled","df","cfi.robust","ifi","rmsea.robust","srmr"))

fitMeasures(structural_model_constrain4,c("chisq.scaled","df","cfi.robust","ifi","rmsea.robust","srmr"))

fitMeasures(structural_model_constrain5,c("chisq.scaled","df","cfi.robust","ifi","rmsea.robust","srmr"))

compareFit(structural_model_constrain3,structural_model_constrain4)
